# Zn(AsO_3_)_2_, a unique structure among arsenates with composition *M*^II^As^V^_2_O_6_

**DOI:** 10.1107/S2056989026009308

**Published:** 2026-09-11

**Authors:** Maximilian Pfeiffer, Berthold Stöger, Matthias Weil

**Affiliations:** a TU Wien, X-Ray Centre, Getreidemarkt 9/E057, 1060 Vienna, Austria; b TU Wien, Institute for Chemical Technologies and Analytics, Division of Applied Solid State Chemistry, Getreidemarkt 9/E164-05-1, 1060 Vienna, Austria; University of Aberdeen, United Kingdom

**Keywords:** crystal structure, structural comparison, isotypism, polyarsenate chain

## Abstract

Zinc bis­[*catena*-arsenate(V)] or *catena*-poly[zinc-di-μ-arsenato(V)-κ^4^*O*:*O*], [Zn(AsO_3_)_2_]_*n*_, is isotypic with the low-temperature modification of Zn(PO_3_)_2_. The asymmetric unit comprises one Zn atom situated on a twofold rotation axis and one As and three O atoms in general positions. In the crystal structure, the principal building units, ZnO_6_ octa­hedra and AsO_4_ tetra­hedra, are condensed into ^1^_∞_[ZnO_4/2_O_2/1_] zigzag chains and (AsO_3_)_2_ polyarsenate chains with a periodicity of two AsO_4_ tetra­hedra, respectively. The two types of chains extend parallel to [001] and are fused into a compact framework structure. Compared with divalent transition metal arsenates and other arsenates of the formula *M*^II^As_2_O_6_, Zn(AsO_3_)_2_ is unique in that arsenic is present exclusively in a tetra­hedral coordination and not in a mixed tetra­hedral/octa­hedral or purely octa­hedral coordination.

## Abstract 2

Zn(AsO_3_)_2_ is isotypic with the low-temperature modification of Zn(PO_3_)_2_ and consists of AsO_4_ tetra­hedra condensed into polyarsenate chains with a periodicity of two tetra­hedra.

## 1. Chemical context

The zinc arsenate Zn(AsO_3_)_2_ has been reported as one of the phases existing in the *pseudo*-binary system ZnO–As_2_O_5_ (Kasenov *et al.*, 1996 ▸). Whereas for the other phases reported in this system complete crystal structure determinations have been carried out in the me­antime (details are given in the database survey), for Zn(AsO_3_)_2_ only unit-cell parameters were given on basis of a powder X-ray diffraction study: crystal system ortho­rhom­bic with *a* = 8.625, *b* = 9.035, *c* = 12.494 Å, *V* = 933 Å^3^ (Mustafin & Kasenov, 1994 ▸, 1996 ▸). Recent results of crystal structure prediction methods claimed the possible existence of Zn(AsO_3_)_2_ (or as stated there of ZnAs_2_O_6_). Whereas one study states that this phase has an existence probability of 0.9105 with a polar structure (Gake *et al.*, 2025 ▸; data given in the supporting information) the other states that it crystallizes in the centrosymmetric PbSb_2_O_6_ structure type with trigonal symmetry (Griesemer *et al.*, 2025 ▸; supplementary material of this article: OQMD ID 1348395).

We sought to investigate these conflicting claims and carried out experiments on the crystal growth of Zn(AsO_3_)_2_. Using a reaction similar to that described by Kasenov *et al.* (1996 ▸), we were able to isolate single crystals of Zn(AsO_3_)_2_ (**I**) and report here on the results of the crystal structure analysis.

## 2. Structural commentary

Our study does not confirm any of the previous statements made regarding the ortho­rhom­bic unit cell or possible crystal structures. Compound (**I**) crystallizes in the monoclinic system in the centrosymmetric space group *C*2/*c* and is isotypic with the corresponding phosphate, *i.e*. low-temperature Zn(PO_3_)_2_ (Averbuch-Pouchot *et al.*, 1983 ▸). The unit cell of Zn(AsO_3_)_2_ reported by Mustafin & Kasenov (1994 ▸, 1996 ▸) has a doubled volume compared to the unit cell determined here, however, with no apparent metrical relation beyond this.

In the crystal structure of Zn(AsO_3_)_2_, the Zn atom is located on a twofold rotation axis (multiplicity 4, Wyckoff letter *e*), and the As and the three O atom sites on general positions (8*f*) of space group *C*2/*c*. The zinc atom in (**I**) exhibits a distorted octa­hedral coordination environment with an average Zn—O bond length of 2.090 Å (Table 1 ▸), in good agreement with the literature value of 2.110 (86) Å derived from 193 [ZnO_6_] coordination polyhedra (Gagné & Hawthorne, 2020 ▸). The [ZnO_6_] octa­hedron shares two of its edges with neighbouring octa­hedra, forming ^1^_∞_[ZnO_4/2_O_2/1_] zigzag-chains extending parallel to [001] (Fig. 1 ▸*a*). The arsenate tetra­hedron condenses into a polyarsenate chain with a periodicity of two tetra­hedra (Fig. 1 ▸*b*). The bond length distribution (Table 1 ▸) in this chain follows the usual pattern, with significantly longer As—O distances to the bridging oxygen atoms (O1, average 1.735 Å) than to the terminal O atoms (O2, O3; average 1.646 Å). The mean average As—O bond length within the AsO_4_ tetra­hedron (1.690 Å) complies with the literature value [1.687 (27) Å from 508 arsenate tetra­hedra; Gagné & Hawthorne, 2018 ▸]. The terminal oxygen atoms of the polyarsenate chain are also the ones that bind to the zinc atoms in adjacent zinc oxide chains, so that the polyarsenate chains are aligned in the same direction as the zinc oxide chains, which leads to a very compact packing in this type of structure (Durif, 1995 ▸). In the crystal structure of (**I**) (Fig. 2 ▸), each zinc oxide chain is surrounded by six polyarsenate chains, and each polyarsenate chain is surrounded by three zinc oxide chains, corresponding to the 1:2 ratio of Zn:As in the formula.

The structure model was confirmed using bond-valence-sum calculations (Brown, 2002 ▸) performed with the *ECoN21* program (Ilinca, 2022 ▸). The calculated bond-valence sums (Table 2 ▸) are close to the expected values of 2, 5 and 2 valence units for Zn, As and O atoms, respectively, and the global instability index (GII) of 0.11 valence units indicates a stable and not particularly strained crystal structure (Salinas-Sanchez *et al.*, 1992 ▸; Brown, 2009 ▸).

Using the *compstru* program (de la Flor *et al.*, 2016 ▸) that is available at the Bilbao Crystallographic Server (Aroyo *et al.*, 2006 ▸), a qu­anti­tative comparison of the isotypic structures of low-temperature Zn(PO_3_)_2_ and Zn(AsO_3_)_2_ was made. The arithmetic mean of the distance between paired atoms is 0.1148 Å, and the absolute distances between paired atoms are 0.0925 Å for Zn1, 0.0598 Å for As1/P1, 0.0901 Å for O1, 0.1146 Å for O2, and the largest distance of 0.2057 Å for O3. The latter O atoms are those which, in both structures, have both the shortest Zn—O and the shortest *X*—O (*X* = As, P) bond lengths. The degree of lattice distortion (0.0264), and the low value for the measure of similarity (0.023) indicate a high degree of similarity for the two crystal structures.

## 3. Database survey

The Inorganic Crystal Structure Database (ICSD, data release 2025.1; Zagorac *et al.*, 2019 ▸) was used for the database search. Known structurally characterized phases in the ZnO–As_2_O_5_ system include the high- and low-temperature modifications of tetra­zinc bis­(arsenate(V)) oxide, Zn_4_(AsO_4_)_2_O (Frerichs *et al.*, 1996*a* ▸), dimorphic trizinc orthoarsenate, Zn_3_(AsO_4_)_2_ (Frerichs *et al.*, 1996*b* ▸), and the pyroarsenate Zn_2_As_2_O_7_ with three modifications existing at different temperatures, one of which is commensurately and one is incommensurately modulated (Weil & Stöger, 2010 ▸).

As already mentioned, Zn(AsO_3_)_2_ is isotypic with Zn(PO_3_)_2_, in its low-temperature modification (Averbuch-Pouchot *et al.*, 1983 ▸). The high-temperature modification of Zn(PO_3_)_2_ can be obtained by quenching from the recrystallized melt and contains a similar polyphosphate chain but in contrast to the low-temperature modification with two unique zinc cations in tetra­hedral coordination (Weil, 2004 ▸). According to the information on the system ZnO–As_2_O_5_ provided by Kasenov *et al.* (1996 ▸), there are no indications of a possible high-temperature form of Zn(AsO_3_)_2_. Other *M*(PO_3_)_2_ polyphosphates isotypic with low-temperature Zn(PO_3_)_2_ and Zn(AsO_3_)_2_ can only be prepared by using high pressure (∼80 kbar) and temperatures (∼1270 K) and are reported for *M* = Ni, Mg, Cu, Co, Fe, Mn, and Cd (Bagieu-Beucher *et al.*, 1976 ▸).

Zn(AsO_3_)_2_ is the only arsenate phase with an *M*:As^V^ ratio of 1:2 where solely tetra­hedral AsO_4_ units are present in the polymeric arsenate anion. Except for *M* = Ba with a *catena*-arsenate anion consisting of AsO_4_ tetra­hedra and AsO_6_ octa­hedra (Weil, 2016 ▸), all other *M*As_2_O_6_ phases crystallize in the PbSb_2_O_6_ type of structure (Hill, 1987 ▸) in the trigonal space group type *P*
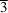
1*m.* This structure type is also referred to as the rosiaite structure type after the mineral of this name (Basso *et al.*, 1996 ▸) and is based on a hexa­gonal array of O atoms with the As atom octa­hedrally coordinated in form of honeycomb layers. This structure type is realized for *M* = Ni, Co, Mn (Nakua & Greedan, 1995 ▸), Cd (Weil, 2001 ▸), Hg (Weil, 2000 ▸), Pd (Orosel & Jansen, 2006 ▸), Ca, Pb (Losilla *et al.*, 1995 ▸), and Sr. The strontium phase is dimorphic and as well as in a rosiaite-type structure also crystallizes in a hexa­gonal superstructure with a doubled unit cell (Weil *et al.*, 2009 ▸).

## 4. Synthesis and crystallization

Crystal growth procedures were carried out in sealed and evacuated silica ampoules using commercially available chemicals as received. A molar 1:1 mixture of ZnO (≥ 99%, Carl Roth GmbH) and As_2_O_5_ [99.9% (metals basis), abcr] was heated at 750 K for about 336 h. X-ray powder diffraction of the bulk product revealed Zn(AsO_3_OH)·H_2_O (mineral name koritnigite; Keller *et al.*, 1980 ▸) and Zn(AsO_3_)_2_ as the main phases in an approximate ratio of 1:2. From this mixture, yellowish crystals of Zn(AsO_3_)_2_ were isolated for the single-crystal X-ray diffraction study. The presence of the hydrous phase Zn(AsO_3_OH)·H_2_O indicates that the hygroscopic starting material As_2_O_5_ was indeed not free of water.

## 5. Refinement

Crystal data, data collection and structure refinement details are summarized in Table 3 ▸. Atomic coordinates and labelling were adapted from the isotypic low-temperature phase of Zn(PO_3_)_2_ (Averbuch-Pouchot *et al.*, 1983 ▸).

## Supplementary Material

Crystal structure: contains datablock(s) I. DOI: 10.1107/S2056989026009308/hb8254sup1.cif

Structure factors: contains datablock(s) I. DOI: 10.1107/S2056989026009308/hb8254Isup2.hkl

CCDC reference: 2585628

Additional supporting information:  crystallographic information; 3D view; checkCIF report

## Figures and Tables

**Figure 1:**
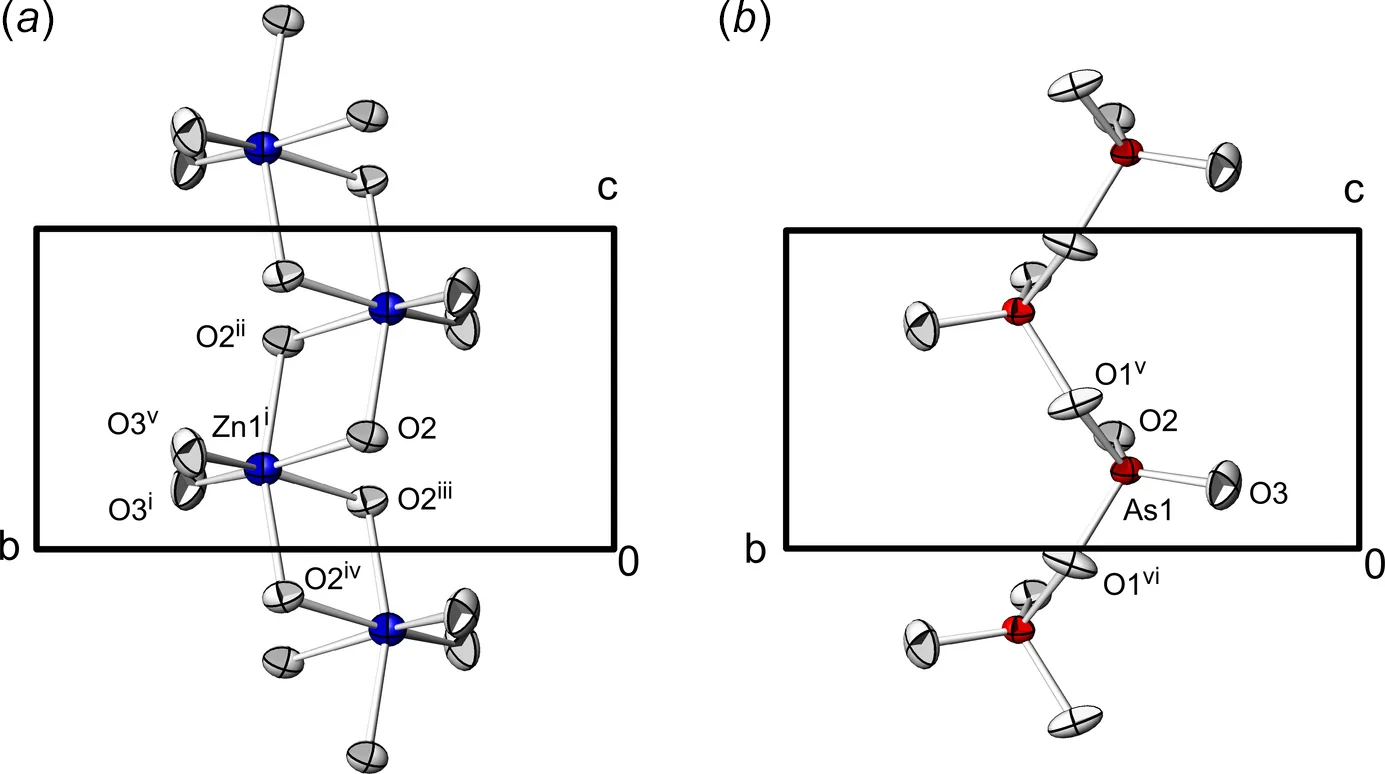
(*a*) One ^1^_∞_[ZnO_4/2_O_2/1_] zigzag chain, and (*b*) one polyarsenate chain crossing the unit cell of (**I**), as shown in projections down [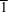
00]. Displacement ellipsoids are drawn at the 97% probability level. [Symmetry codes: (i) *x* + 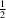, *y* + 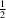, *z*; (ii) −*x* + 1, −*y* + 1, −*z* + 1; (iii) −*x* + 1, *y*, −*z* + 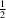; (iv) *x*, −*y* + 1, *z* − 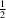; (v) −*x* + 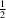, *y* + 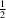, −*z* + 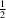; (vi) −*x* + 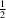, −*y* + 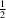, −*z*.]

**Figure 2:**
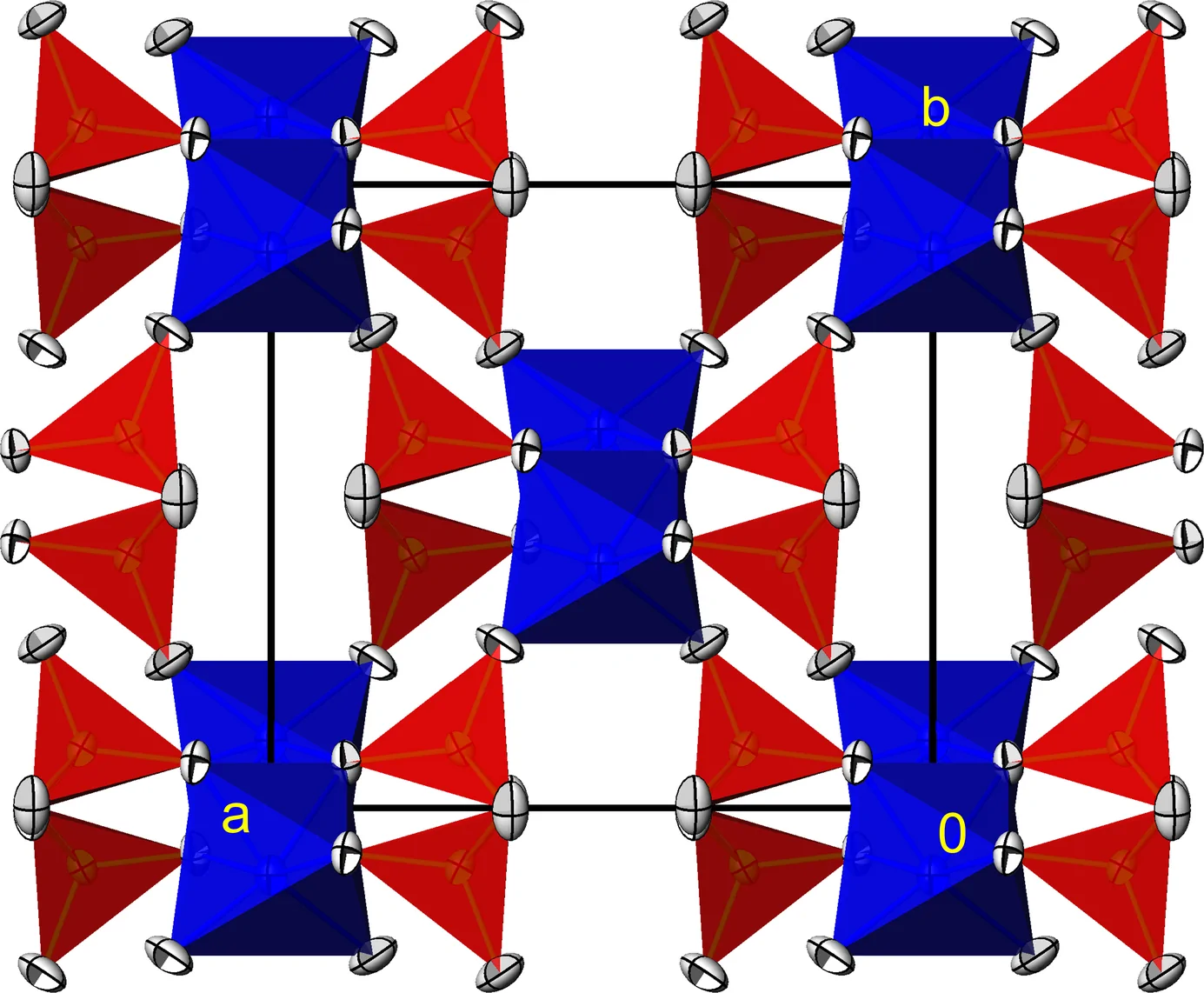
The crystal structure of (**I**) in polyhedral representation (ZnO_6_ octa­hedra blue, AsO_4_ tetra­hedra red), viewed down [00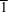]. Displacement ellipsoids are drawn at the 97% probability level.

**Table 1:** Selected bond lengths (Å)

Zn1—O3	2.0276 (10)	As1—O2	1.6518 (10)
Zn1—O2^i^	2.0786 (10)	As1—O1^iii^	1.7319 (10)
Zn1—O2^ii^	2.1640 (10)	As1—O1^iv^	1.7379 (10)
As1—O3	1.6336 (10)		

Symmetry codes: (i) 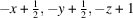; (ii) 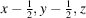; (iii) 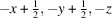; (iv) 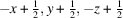.

**Table 2:** Bond-valence-sum calculations (in valence units) for Zn(AsO_3_)_2_

	Zn1	As1	Σ
O1		1.10, 1.08	2.18
O2	0.36 (1×→, 2×↓), 0.29 (1×→, 2×↓)	1.38	2.03
O3	0.41 (1×→, 2×↓)	1.45	1.86
Σ	2.12	5.01	

**Table 3:** Experimental details

Crystal data	
Chemical formula	[Zn(AsO_3_)_2_]
*M*_r_	311.21
Crystal system, space group	Monoclinic, *C*2/*c*
Temperature (K)	300
*a*, *b*, *c* (Å)	10.1061 (10), 9.0308 (8), 5.2801 (5)
β (°)	108.374 (7)
*V* (Å^3^)	457.33 (8)
*Z*	4
Radiation type	Mo *K*α
μ (mm^−1^)	19.68
Crystal size (mm)	0.04 × 0.02 × 0.01

Data collection	
Diffractometer	Stoe Stadivari
Absorption correction	Multi-scan (*LANA*; Koziskova *et al.*, 2016 ▸)
*T*_min_, *T*_max_	0.492, 0.654
No. of measured, independent and observed [*I* > 2σ(*I*)] reflections	3846, 797, 770
*R*_int_	0.017
(sin θ/λ)_max_ (Å^−1^)	0.762

Refinement	
*R*[*F*^2^ > 2σ(*F*^2^)], *wR*(*F*^2^), *S*	0.010, 0.028, 1.13
No. of reflections	797
No. of parameters	43
Δρ_max_, Δρ_min_ (e Å^−3^)	0.40, −0.37

Computer programs: *X-AREA* (Folkers-Karlsson *et al.*, 2026 ▸), *SHELXL* (Sheldrick, 2015 ▸), *OLEX2* (Dolomanov *et al.*, 2009 ▸), *ATOMS* (Dowty, 2006 ▸) and *publCIF* (Westrip, 2010 ▸).

## supplementary crystallographic information

### catena-Poly[zinc-di-µ-arsenato(V)-κ4O:O] Crystal data

**Table:**

[Zn(AsO_3_)_2_]	*F*(000) = 576
*M**_r_* = 311.21	*D*_x_ = 4.520 Mg m^−^^3^
Monoclinic, *C*2/*c*	Mo *K*α radiation, λ = 0.71073 Å
*a* = 10.1061 (10) Å	Cell parameters from 5088 reflections
*b* = 9.0308 (8) Å	θ = 3.1–32.8°
*c* = 5.2801 (5) Å	µ = 19.68 mm^−^^1^
β = 108.374 (7)°	*T* = 300 K
*V* = 457.33 (8) Å^3^	Shard, yellow
*Z* = 4	0.04 × 0.02 × 0.01 mm

### catena-Poly[zinc-di-µ-arsenato(V)-κ4O:O] Data collection

**Table:**

Stoe Stadivari diffractometer	797 independent reflections
Radiation source: Axo_Mo	770 reflections with *I* > 2σ(*I*)
Graded multilayer mirror monochromator	*R*_int_ = 0.017
Detector resolution: 13.33 pixels mm^-1^	θ_max_ = 32.8°, θ_min_ = 3.1°
rotation method, ω scans	*h* = −14→15
Absorption correction: multi-scan (*LANA*; Koziskova *et al.*, 2016)	*k* = −12→13
*T*_min_ = 0.492, *T*_max_ = 0.654	*l* = −7→4
3846 measured reflections	

### catena-Poly[zinc-di-µ-arsenato(V)-κ4O:O] Refinement

**Table:**

Refinement on *F*^2^	Primary atom site location: isomorphous structure methods
Least-squares matrix: full	*w* = 1/[σ^2^(*F*_o_^2^) + (0.0127*P*)^2^ + 0.509*P*] where *P* = (*F*_o_^2^ + 2*F*_c_^2^)/3
*R*[*F*^2^ > 2σ(*F*^2^)] = 0.010	(Δ/σ)_max_ = 0.001
*wR*(*F*^2^) = 0.028	Δρ_max_ = 0.40 e Å^−^^3^
*S* = 1.13	Δρ_min_ = −0.37 e Å^−^^3^
797 reflections	Extinction correction: SHELXL (Sheldrick, 2015), Fc^*^=kFc[1+0.001xFc^2^λ^3^/sin(2θ)]^-1/4^
43 parameters	Extinction coefficient: 0.0035 (2)
0 restraints	

### catena-Poly[zinc-di-µ-arsenato(V)-κ4O:O] Special details

**Table:**

Geometry. All esds (except the esd in the dihedral angle between two l.s. planes) are estimated using the full covariance matrix. The cell esds are taken into account individually in the estimation of esds in distances, angles and torsion angles; correlations between esds in cell parameters are only used when they are defined by crystal symmetry. An approximate (isotropic) treatment of cell esds is used for estimating esds involving l.s. planes.

### catena-Poly[zinc-di-µ-arsenato(V)-κ4O:O] Fractional atomic coordinates and isotropic or equivalent isotropic displacement parameters (Å^2^)

**Table:**

	*x*	*y*	*z*	*U*_iso_*/*U*_eq_	
Zn1	0.000000	0.10801 (2)	0.250000	0.00841 (6)	
As1	0.21468 (2)	0.40539 (2)	0.24523 (3)	0.00640 (5)	
O1	0.36157 (10)	−0.00460 (12)	0.04492 (19)	0.0121 (2)	
O2	0.38566 (10)	0.42724 (11)	0.35206 (19)	0.00870 (17)	
O3	0.15428 (11)	0.23634 (11)	0.1990 (2)	0.0132 (2)	

### catena-Poly[zinc-di-µ-arsenato(V)-κ4O:O] Atomic displacement parameters (Å^2^)

**Table:**

	*U*^11^	*U*^22^	*U*^33^	*U*^12^	*U*^13^	*U*^23^
Zn1	0.00662 (11)	0.00985 (11)	0.00828 (10)	0.000	0.00168 (8)	0.000
As1	0.00579 (7)	0.00774 (8)	0.00560 (7)	−0.00148 (4)	0.00169 (5)	−0.00034 (4)
O1	0.0078 (4)	0.0209 (5)	0.0074 (4)	0.0001 (4)	0.0021 (3)	0.0056 (4)
O2	0.0057 (4)	0.0118 (4)	0.0077 (4)	−0.0008 (3)	0.0008 (3)	0.0008 (3)
O3	0.0139 (5)	0.0094 (4)	0.0172 (5)	−0.0052 (4)	0.0061 (4)	−0.0020 (4)

### catena-Poly[zinc-di-µ-arsenato(V)-κ4O:O] Geometric parameters (Å, º)

**Table:**

Zn1—O3	2.0276 (10)	Zn1—O2^v^	2.1640 (10)
Zn1—O3^i^	2.0276 (10)	As1—O3	1.6336 (10)
Zn1—O2^ii^	2.0786 (10)	As1—O2	1.6518 (10)
Zn1—O2^iii^	2.0786 (10)	As1—O1^vi^	1.7319 (10)
Zn1—O2^iv^	2.1640 (10)	As1—O1^vii^	1.7379 (10)
			
O3—Zn1—O3^i^	110.28 (6)	O2^iii^—Zn1—O2^v^	88.05 (3)
O3—Zn1—O2^ii^	99.15 (4)	O2^iv^—Zn1—O2^v^	82.06 (5)
O3^i^—Zn1—O2^ii^	90.92 (4)	O3—As1—O2	117.65 (5)
O3—Zn1—O2^iii^	90.92 (4)	O3—As1—O1^vi^	108.08 (5)
O3^i^—Zn1—O2^iii^	99.15 (4)	O2—As1—O1^vi^	111.48 (5)
O2^ii^—Zn1—O2^iii^	162.38 (6)	O3—As1—O1^vii^	108.20 (5)
O3—Zn1—O2^iv^	163.24 (4)	O2—As1—O1^vii^	110.65 (5)
O3^i^—Zn1—O2^iv^	84.54 (4)	O1^vi^—As1—O1^vii^	99.15 (3)
O2^ii^—Zn1—O2^iv^	88.05 (3)	As1^vi^—O1—As1^v^	130.01 (6)
O2^iii^—Zn1—O2^iv^	78.64 (4)	As1—O2—Zn1^iii^	121.02 (5)
O3—Zn1—O2^v^	84.54 (4)	As1—O2—Zn1^viii^	126.04 (5)
O3^i^—Zn1—O2^v^	163.24 (4)	Zn1^iii^—O2—Zn1^viii^	101.35 (4)
O2^ii^—Zn1—O2^v^	78.64 (4)	As1—O3—Zn1	141.36 (6)

Symmetry codes: (i) −*x*, *y*, −*z*+1/2; (ii) *x*−1/2, −*y*+1/2, *z*−1/2; (iii) −*x*+1/2, −*y*+1/2, −*z*+1; (iv) *x*−1/2, *y*−1/2, *z*; (v) −*x*+1/2, *y*−1/2, −*z*+1/2; (vi) −*x*+1/2, −*y*+1/2, −*z*; (vii) −*x*+1/2, *y*+1/2, −*z*+1/2; (viii) *x*+1/2, *y*+1/2, *z*.
